# Nucleomorph Genome Sequences of Two Chlorarachniophytes, *Amorphochlora amoebiformis* and *Lotharella vacuolata*

**DOI:** 10.1093/gbe/evv096

**Published:** 2015-05-22

**Authors:** Shigekatsu Suzuki, Shu Shirato, Yoshihisa Hirakawa, Ken-Ichiro Ishida

**Affiliations:** ^1^Graduate School of Life and Environmental Sciences, University of Tsukuba, Ibaraki, Japan; ^2^Faculty of Life and Environmental Sciences, University of Tsukuba, Ibaraki, Japan

**Keywords:** chlorarachniophyte, nucleomorph, endosymbiosis, genome reduction, secondary plastid

## Abstract

Many algal groups acquired complex plastids by the uptake of green and red algae through multiple secondary endosymbioses. As a result of gene loss and transfer during the endosymbiotic processes, algal endosymbiont nuclei disappeared in most cases. However, chlorarachniophytes and cryptophytes still possess a relict nucleus, so-called the nucleomorph, of the green and red algal endosymbiont, respectively. Nucleomorph genomes are an interesting and suitable model to study the reductive evolution of endosymbiotically derived genomes. To date, nucleomorph genomes have been sequenced in four cryptophyte species and two chlorarachniophyte species, including *Bigelowiella natans* (373 kb) and *Lotharella oceanica* (610 kb). In this study, we report complete nucleomorph genome sequences of two chlorarachniophytes, *Amorphochlora amoebiformis* and *Lotharella vacuolata*, to gain insight into the reductive evolution of nucleomorph genomes in the chlorarachniophytes. The nucleomorph genomes consist of three chromosomes totaling 374 and 432 kb in size in *A. amoebiformis* and *L. vacuolata*, respectively. Comparative analyses among four chlorarachniophyte nucleomorph genomes revealed that these sequences share 171 function-predicted genes (86% of total 198 function-predicted nucleomorph genes), including the same set of genes encoding 17 plastid-associated proteins, and no evidence of a recent nucleomorph-to-nucleus gene transfer was found. This suggests that chlorarachniophyte nucleomorph genomes underwent most of their reductive evolution prior to the radiation of extent members of the group. However, there are slight variations in genome size, GC content, duplicated gene number, and subtelomeric regions among the four nucleomorph genomes, suggesting that the genomes might be undergoing changes that do not affect the core functions in each species.

## Introduction

Plastids were acquired through several endosymbiotic events between a heterotrophic eukaryote and a photosynthetic organism. Plants and some algae (glaucophytes, green, and red algae) acquired plastids through a single primary endosymbiosis between a eukaryote and a cyanobacterium (Rodríguez-Ezpeleta et al. 2005; Price et al. 2012). The other algal groups (chlorarachniophytes, cryptophytes, dinoflagellates, euglenophytes, haptophytes, and heterokonts) and a nonphotosynthetic parasite group (apicomplexans) have complex plastids that originated by the uptake of green and red algal endosymbionts through multiple secondary endosymbioses (Ishida 2005; Gould et al. 2008; Archibald 2009; Keeling 2010). These primary and secondary endosymbioses resulted in the remarkable diversity of photosynthetic eukaryotes found across the tree of life.

Chlorarachniophytes acquired complex plastids by the ingestion of a green algal endosymbiont. The endosymbiont is closely related to the ulvophyceae–trebouxiophyceae–chlorophyceae clade, and the host is a cercozoan like the small predator *Minorisa minuta* of the Rhizaria supergroup (Ishida et al. 1997, 1999; Rogers et al. 2007; del Campo et al. 2012). This algal group is of special interest because their complex plastids still harbor a relict nucleus of the endosymbiont, which has disappeared in most cases of secondary endosymbioses (Palmer 1997). The relic nucleus, so-called the nucleomorph, is localized in the periplastidal compartment, the space between the inner and outer pair of plastid membranes, which is the remnant cytoplasm of the endosymbiont (Hibberd and Norris 1984). During the secondary endosymbiosis, nuclear genes of integrated green alga were lost in massive numbers and transferred to host nuclear genomes; thus, the nucleomorph genome has been extremely reduced in size. Interestingly, nucleomorphs have also been found in cryptophyte plastids that originated from a red algal endosymbiont (Douglas et al. 1991; Douglas and Penny 1999). Therefore, two distant algal groups evolved highly reduced nucleomorph genomes through different routes from different starting points. Nucleomorph genomes offer an interesting opportunity to study the reductive evolution of endosymbiotically derived genomes.

To date, nucleomorph genomes have been sequenced in two chlorarachniophytes, *Bigelowiella natans* (Gilson et al. 2006) and *Lotharella oceanica* (Tanifuji et al. 2014), and four cryptophytes, *Guillardia theta* (Douglas et al. 2001), *Hemiselmis andersenii* (Lane et al. 2007), *Cryptomonas paramecium* (Tanifuji et al. 2011), and *Chroomonas mesostigmatica* (Moore et al. 2012). Comparative investigations have revealed that the nucleomorph genomes share many conserved features even between chlorarachniophytes and cryptophytes (Archibald 2007; Archibald and Lane 2009). For instance, all of the nucleomorph genomes are composed of three small chromosomes, which generally possess ribosomal DNA (rDNA) operons in the subtelomeric regions at both ends. Recently, polyploidy of nucleomorph genomes has been reported in *B. natans* (diploid) and *G. theta* (tetraploid) (Hirakawa and Ishida 2014). The nucleomorph genomes (373–703 kb in size) tightly encode only 284–610 proteins. Many genes encode housekeeping proteins for eukaryotic core functions (e.g., translation, transcription, and protein folding) and others are nucleomorph-specific orphan genes (ORFans) that encode hypothetical proteins showing no sequence similarity to any known proteins. Interestingly, conserved sets of plastid-associated proteins were found to be encoded by nucleomorph genomes. For example, 17 proteins are shared in the two chlorarachniophytes, and 31 proteins are shared in three cryptophytes, excluding the nonphotosynthetic *Cr. paramecium*. However, nucleomorph-encoded genes are insufficient to maintain the nucleomorph function, and all nucleomorph genomes sequenced so far lack DNA polymerase genes. Recently, the nucleomorph genomes of *B. natans* and *G. theta* were sequenced, which revealed that over 1,000 nucleus-encoded proteins were presumed to be targeted to periplastidal compartments to compensate for nucleomorph lacking genes (Curtis et al. 2012). As a notable feature, a massive number of ultrasmall introns has been found in chlorarachniophyte nucleomorph genes despite the extreme compaction of nucleomorph genomes. *Bigelowiella natans* and *L. oceanica* nucleomorph genomes have 852 and 1,011 introns, respectively, ranging from 18 to 23 nucleotides (nt), with typical spliceosomal GT–AG boundaries (Gilson et al. 2006; Tanifuji et al. 2014).

Pulsed-field gel electrophoresis (PFGE) analyses have revealed that the nucleomorph genomes vary in size, and the predicted nucleomorph genome sizes of chlorarachniophytes and cryptophytes range from 330 to 1,033 kb and from 495 to 750 kb, respectively (Eschbach et al. 1991; Rensing et al. 1994; Gilson and McFadden 1999; Lane and Archibald 2006; Silver et al. 2007; Phipps et al. 2008; Tanifuji et al. 2010). Several factors that contribute to the size variation of cryptophyte nucleomorph genomes have been identified (Lane et al. 2007; Tanifuji et al. 2011; Moore et al. 2012). The average length of protein-coding genes and the total number of genes are slightly different among the four cryptophyte nucleomorph genomes sequenced so far, and the most remarkable difference is found in the length of intergenic spacers. A comparison of chlorarachniophyte nucleomorph genomes between *B. natans* (373 kb) and *L. oceanica* (610 kb) revealed that the size variation is mainly caused by multiple duplicated genes (Tanifuji et al. 2014).

To gain further insight into nucleomorph genome evolutionary processes in chlorarachniophytes, we sequenced the nucleomorph genomes of two different species, *Amorphochlora amoebiformis* and *Lotharella vacuolata*. *Lotharella vacuolata* is closely related to *L. oceanica*, and *A. amoebiformis* belongs to a phylogenetically distinct genus. The nucleomorph genomes of *A. amoebiformis* and *L. oceanica* are 374 and 432 kb in size, respectively. Our comparative analyses of four chlorarachniophyte nucleomorph genomes indicate that all sequences share 189 protein-coding genes, including the same set of genes encoding 17 plastid-associated proteins. The most remarkable difference among the four genomes was the existence of multiple duplicated regions across the nucleomorph genomes of *Lotharella* species, which mainly caused the variation in the size of nucleomorph genomes. Our results suggest that chlorarachniophyte nucleomorph genomes have reached an end point in reductive evolution, whereas the increases in genome size occurred in some species individually.

## Materials and Methods

### Nucleomorph DNA Extraction and Sequencing

*Amorphochlora amoebiformis* (CCMP2058) and *L. vacuolata* (CCMP240) were cultured at 20 °C under white light conditions (80–100 μmol photons·m^−^^2 ^s^−^^2^) on a 12:12 h light:dark cycle in ESM medium (Kasai et al. 2009). Cells were collected by general centrifugation from 2- to 3-week-old cultures. Nucleomorph DNA was separated by PFGE, according to the conditions outlined by Silver et al. (2007). The separated nucleomorph DNA was purified from the gel slice by electroelution with dialysis membrane tubing (Moore et al. 2002). Shotgun libraries were generated and Sanger sequenced at the National Institution of Genetics in Japan. Additional sequencing of the *L. vacuolata* nucleomorph genome was carried out through the 454 GS Junior System (454 Life Sciences; a Roche Co., Branford, CT) with DNA extracted from isolated plastids. *Lotharella vacuolata* cells were resuspended in 10 ml of modified isolation buffer (600 mM d-Sorbitol, 10 mM KCl, 5 mM ethylenediaminetetraacetic acid [EDTA], 1 mM MgCl_2_, 1 mM MnCl_2_, and 50 mM HEPES-KOH, pH 7.6) (Hopkins et al. 2012) and disrupted by a Yeda press with 60 kg cm^−^^2^ pressure at 4 °C. The resulting sample was loaded in a Percoll step gradient (20%, 30%, and 40% in gradient buffer containing 600 mM d-Sorbitol, 5 mM EDTA, and 50 mM HEPES-KOH, pH 7.6) and centrifuged at 3,300 × g for 20 min at 4 °C. Plastids were enriched in interphase between 20% and 30%, and DNA was extracted from this fraction, using the CTAB, cetyltrimethylammonium bromide method (Ishida et al. 1999).

### Genome Assembly and Annotation

In total, 13,734 (10,174,889 bp) and 33,256 (24,900,190 bp) Sanger reads of *A. amoebiformis* and *L. vacuolata* were assembled using CodonCode Aligner (CodonCode Co., Centerville, MA), respectively. A total of 105,915 reads (44,084,934 bp) of *L. vacuolata* from the 454 GS Junior System were assembled using Newbler Assembler v. 2.5 (454 Life Sciences, a Roche Co.). The Sanger *L. vacuolata* contigs were reassembled with the 454 GS Junior contigs by using CodonCode Aligner. In total, 17 and 56 resulting nucleomorph contigs of *A. amoebiformis* and *L. vacuolata* were obtained, respectively, and gaps were filled by multiple polymerase chain reactions (PCR) with 14 and 53 sets of primers, respectively. To confirm the sequences of duplicated gene regions in the *L. vacuolata* nucleomorph genome, we amplified those regions by PCR and sequenced them with the ABI 3130 Genetic Analyzer (Applied Biosystems, Life Technologies, Carlsbad, CA).

We manually identified open reading flames (ORFs) longer than 50 amino acids in the nucleomorph genomes using the Artemis Genome Browser 13.2.0 (Rutherford et al. 2000). Ultrasmall introns were initially assumed to be 18–23 nt with a typical spliceosomal boundary (5′-GT and AG-3′) based on the chlorarachniophyte nucleomorph genes sequenced so far. To presume the function of protein-coding genes, we performed homology searches with BLASTx and BLASTp against sequence databases in National Center for Biotechnology Information (Altschul et al. 1997) with a cutoff *e* value of 0.001. Based on the BLAST (Basic Local Alignment Search Tool) surveys, ORFs coding hypothetical proteins that have no similarity with any sequences in other organisms are defined as orphan genes (ORFans). rRNAs were identified using RNAmmer 1.2 (Lagesen et al. 2007) and BLASTn against rRNA sequences of *B. natans*. Transfer RNAs (tRNAs) and permuted tRNAs were predicted by tRNAscan-SE v. 2.1 (Schattner et al. 2005) and SPLITS (Sugahara et al. 2006), and the following parameters were applied: -c -p 0.55 -F -3 -h -3 (Soma et al. 2007) and -c -p 0.6 -F -1 (Maruyama et al. 2010). Small nuclear RNAs (snRNAs) were detected using fRNAdb with an option (word size = 7) (Kin et al. 2007). Simple repeat sequences in nucleomorph genomes were identified by the RepeatMasker (http://www.repeatmasker.org/, last accessed June 2, 2015). For comparative analyses, we also reconsidered ORFs of *B. natans* and *L. oceanica* and altered the number of protein-coding genes and introns (table 1). Nucleomorph genome sequences of *A. amoebiformis* and *L. vacuolata* were deposited in GenBank/DDBJ/EMBL, and the accession numbers are AB996602–AB996604 and AB996599–AB996601, respectively.

**Table 1:** Genome Features of Nucleomorph Genomes in Chlorarachniophytes

	*Amorphochlora amoebiformis*	*Lotharella vacuolata*	*Bigelowiella natans*^a^	*Lotharella oceanica*^b^
Genome size (bp)	373,958	431,876	372,879	∼611,658
Chr. 1	131,920	166,173	140,598	∼210,000
Chr. 2	124,024	141,647	134,144	207,543
Chr. 3	118,014	124,056	98,137	194,115
GC content (%)	30.0	24.7	28.5	33.0
Number of genes	340	359	332	636
Protein-coding (including duplicates)	295 (300)	294 (319)	288 (288)	338 (596)
rRNAs	3 (18)	3 (18)	3 (18)	3 (18)
tRNAs	21	19	22	19
snRNAs	3	3	4	3
Introns (introns/genes)	793 (2.6)	1,028 (3.2)	865 (3.0)	1,021 (1.6)
Gene density (genes/kb)	0.91	0.83	0.89	1.04

^a^They were updated from the original article (Gilson et al. 2006).

^b^They were updated from the original article (Tanifuji et al. 2014).

### Comparative Analyses

In total, 188 of the shared proteins were used to calculate the average size of nucleomorph-encoded proteins. To determine the statistical significance of size differences in gene-coding and intergenic regions among nucleomorph genomes, we employed a one-way analysis of variance (ANOVA) with StatPlus:mac (http://www.analystsoft.com/en/, last accessed June 2, 2015). Homologous genes among four nucleomorph genomes of chlorarachniophytes were searched using MCScanX (Wang et al. 2012), based on their amino acid sequence homology (*e* value < 0.001) (listed in supplementary table S1, Supplementary Material online). Positions of homologous genes were manually compared among nucleomorph chromosomes, which are shown in line images created by MCScanX. Syntenic blocks consisting of at least four homologous genes in the same order were identified using the same definition as that used by Moore et al. (2012).

To examine the possibility of a recent gene transfer from the nucleomorph to the nucleus after the divergence of chlorarachniophyte species, we searched nuclear genes for genes missing from individual nucleomorph genomes. Seven and 18 genes were absent from the *B. natans* and *A. amoebiformis* nucleomorph genomes compared with the other three nucleomorph genomes, respectively. These genes were searched in the nuclear genome of *B. natans* (Curtis et al. 2012) or in the nuclear transcriptome of *A. amoebiformis* by BLASTx with a cutoff *e* value of 1E^−^^5^. The *A. amoebiformis* RNA-seq transcriptome data were generated by the National Center for Genome Resources as a part of the Marine Microbial Eukaryotic Transcriptome Sequencing Project (Keeling et al. 2014) (the sample ID is MMETSP0042).

## Results and Discussion

### Architectures of Two Nucleomorph Genomes in Chlorarachniophytes

**Fig. 1.—:**
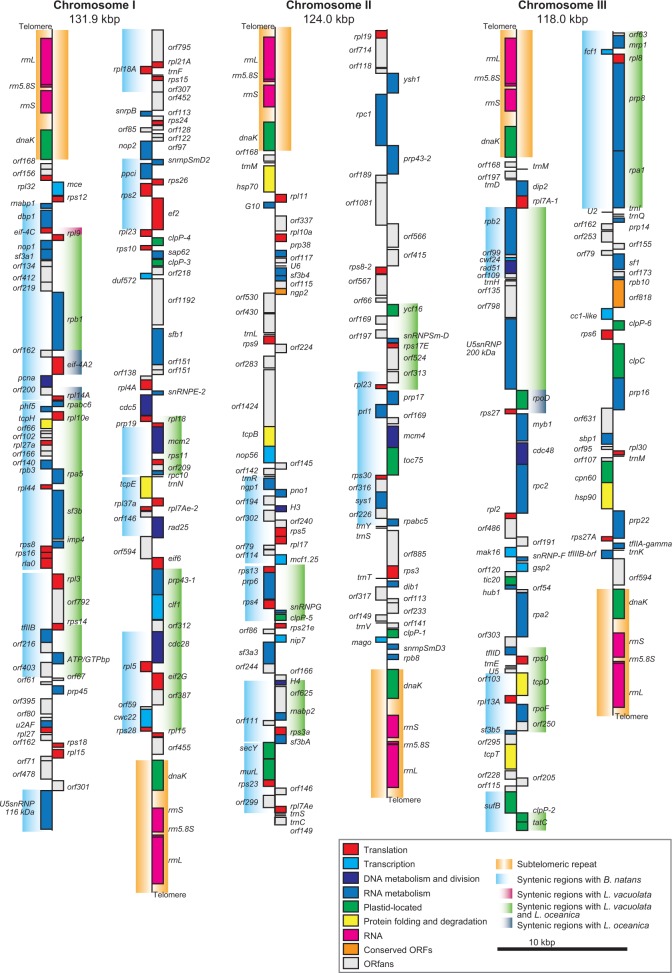
Nucleomorph genome map of the chlorarachniophyte *A. amoebiformis*. The genome is comprised three chromosomes, which are shown as being artificially broken at their midpoint. Genes indicated on the right side are transcribed from top to bottom, and the genes on the left side are transcribed in the opposite direction. Colors of gene blocks correspond to predicted functional categories in the box. Syntenic regions with *B. natans* (blue), *L. vacuolata* (red), *L. oceanica* (gray), and both *L. vacuolata* and *L. oceanica* (green) are shaded by color gradations.

**Fig. 2.—:**
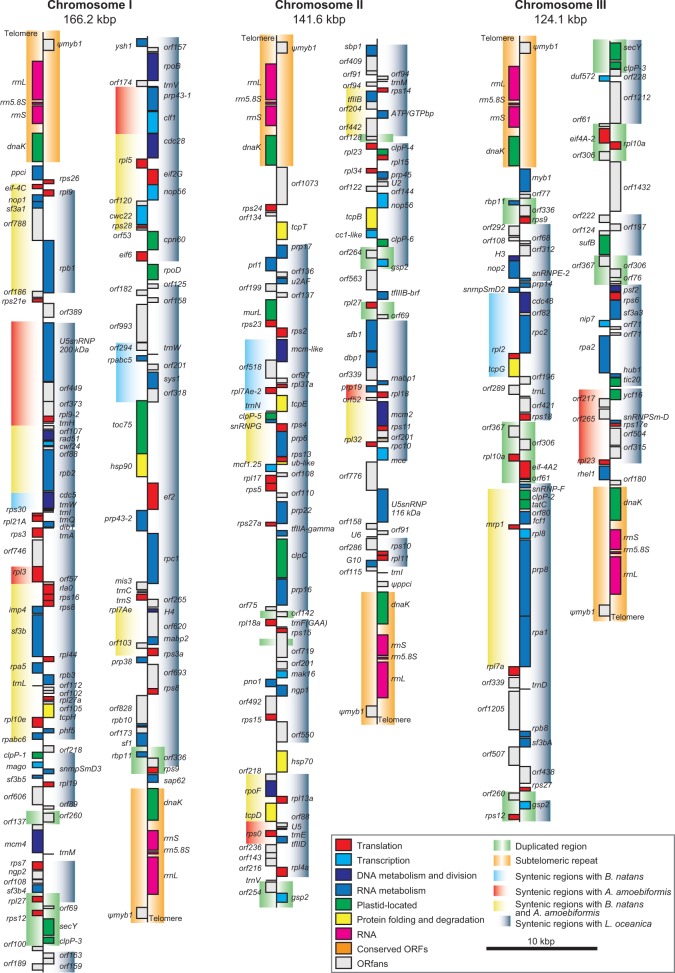
Nucleomorph genome map of the chlorarachniophyte *L. vacuolata*. The genome is comprised three chromosomes, which are shown artificially broken at their midpoint. Genes indicated on the right side are transcribed from top to bottom, and the genes on the left side are transcribed in the opposite direction. Colors of gene blocks correspond to predicted functional categories in the box. Duplicated gene regions (green) and syntenic regions with *B. natans* (blue), *A. amoebiformis* (red), *L. oceanica* (gray), and both *B. natans* and *A. amoebiformis* (yellow) are shaded by color gradations.

The general characteristics of nucleomorph genomes in *A. amoebiformis* and *L. vacuolata* are summarized in table 1. Both nucleomorph genomes are composed of three chromosomes totaling 374.0 kb (131.9, 124.0, and 118.0 kb) in *A. amoebiformis* and 431.9 kb (166.2, 141.6, and 124.1 kb) in *L. vacuolata*. The actual genome sizes were slightly different from the predicted sizes, according to the PFGE analyses, approximately 330 and approximately 450 kb (Silver et al. 2007). The GC content is 30.0% and 24.7% in *A. amoebiformis* and *L. vacuolata*, respectively. The number of total genes is predicted to be 340, including 300 protein-coding genes, 21 tRNAs, 18 rRNAs, and 3 noncoding RNAs (ncRNAs) in the *A. amoebiformis* nucleomorph genome, and 359 genes, including 319 protein-coding genes, 19 tRNAs, 18 rRNAs, and 3 ncRNAs, in *L. vacuolata* (table 1). The gene density is 0.91 and 0.83 genes/kb in each nucleomorph genome (table 1). All three chromosomes of *A. amoebiformis* and *L. vacuolata* carry identical sequences in the six subtelomeric regions comprised an rDNA operon (SSU rDNA, 5.8S rDNA, and LSU rDNA) and the *dnaK* gene (figs. 1 and 2). When these genomes were compared with the nucleomorph genomes of two other chlorarachniophytes, *B. natans* and *L. oceanica*, all four nucleomorph genomes generally showed similar architectures; however, several notable variations were found.

The most remarkable difference is in the genome sizes. The *L. vacuolata* nucleomorph genome is approximately 50 kb larger than those of *A. amoebiformis* and *B. natans* (table 1). The primary factor leading to this size variation is the existence of multiple duplicated genes spreading in the *L. vacuolata* nucleomorph genome. Although no duplicated gene exists in the *A. amoebiformis* and *B. natans* nucleomorph genomes, excluding the subtelomeric repeats, *L. vacuolata* has 13 duplicated regions, including 35 complete and 7 partial genes, totaling 37.8 kb in size (fig. 2 and table 2). The duplicated gene sequences were exactly identical, and 12 and 23 of those genes have intra- and interchromosomal copies, respectively. Interestingly, multiple gene duplications have also been found in the large nucleomorph genome of *L. oceanica* (∼612 kb) (Tanifuji et al. 2014) that is closely related to *L. vacuolata*, and some of the duplicated genes (e.g., *rpl27*, *rpl12*, *secY*, *gsp2*, and *clpP-3*) are shared between these two *Lotharella* species (fig. 2). This suggests that the genome size increase occurred through gene duplication before the divergence of *Lotharella* species. Furthermore, the *L. oceanica* nucleomorph genome carries a long subtelomeric sequence consisting of 45 ORFs between the SSU rDNA and *dnaK* of all six chromosome ends (total ∼210 kb) (Tanifuji et al. 2014), which is not seen in *L. vacuolata*. The *L. oceanica* nucleomorph genome appears to have acquired these subtelomeric sequences after the divergence of these two species. The length of intergenic regions also contributes the size variation of the chlorarachniophyte nucleomorph genomes. The average length of intergenic regions is 134.5 bp (*n* = 356), 163.0 bp (*n* = 633), 112.5 (*n* = 329), and 86.6 (*n* = 339) in *L. vacuolata*, *L. oceanica*, *B. natans*, and *A. amoebiformis*, respectively, which are significantly different (*P* < 0.001, ANOVA) (table 2). It has been reported that cryptophyte nucleomorph genomes also have size variations, and multiple duplicated genes and slightly longer intergenic spacers contribute to increases in the size of the relatively large nucleomorph genome in *Ch. mesostigmatica* (Moore et al. 2012). Interestingly, similar factors contribute to the size variation of nucleomorph genomes in both chlorarachniophytes and cryptophytes, despite their independent origins.

**Table 2:** Genome Size Variation and Its Factors

	*Amorphochlora amoebiformis*	*Lotharella vacuolata*	*Bigelowiella natans*	*Lotharella oceanica*
Genome size (kb)	374.0	431.9	372.9	∼611.7
Average size of shared proteins (aa)^a^	346.8	355.7	347.3	351.0
Intergenic length (bp)^b^	86.6	130.4	112.5	163.0
Size of duplicated region (kb)	63.6	109.2	52.4	282.7
Internal regions	0.0	37.8	0.0	54.5
Subtelomeric regions	63.6	71.4	52.4	228.2

^a^Variations are not significantly shown by ANOVA (*P* > 0.05).

^b^Variations are significantly shown by ANOVA (*P* ≤ 0.01).

The telomeric and subtelomeric regions were found to have slight variations among the chlorarachniophyte nucleomorph genomes. The telomere sequence of *A. amoebiformis* is composed of [TCCTGGG] repeats, whereas other species typically carry [TCTAGGG]n. Moreover, the typical telomere sequence of chlorophytes is [TTTAGGG]n (Fulnečková et al. 2012), suggesting that an ancestral chlorarachniophyte had telomeric repeats of [TCTAGGG], and *A. amoebiformis* acquired the substitutions in the telomeric sequence. Subtelomeric regions consisted of an rDNA operon (SSU rDNA, 5.8S rDNA, and LSU rDNA), which is highly conserved in all nucleomorph genomes; however, there is variation in the gene order. The *A. amoebiformis*, *L. vacuolata*, and *L. oceanica* nucleomorph genomes carry the sequence of SSU–5.8S–LSU–telemere in this order, whereas the *B. natans* and *Chlorarachnion reptans* rDNA operons lie in the opposite direction (LSU–5.8S–SSU–telemere) (Silver et al. 2010). An inversion event of the rDNA operon is assumed to have occurred in a common ancestor shared between *B. natans* and *Chl. reptans,* based on phylogeny (Silver et al. 2010). Pseudogenes that partially encode the 3′-end of *myb1* were found to reside on each of the LSU rDNA downstream regions in *L. vacuolata* (fig. 2), and similar *dnaK* pseudogenes exist in the LSU rDNA downstream regions in *B. natans* (Gilson et al. 2006). The functional *myb1* and *dnaK* genes are located near the subtelomeric region in one of the *L. vacuolata* and *B. natans* chromosomes, respectively. These pseudogenes would be unexpected products of interchromosomal recombination because nucleomorph subtelomeric regions are thought to have undergone frequent gene conversions through interchromosomal recombination to maintain the nearly identical rDNA sequences (Tanifuji et al. 2014).

### Gene Content of Nucleomorph Genomes

Similar numbers of tRNAs, rRNAs, and ncRNAs are found in the chlorarachniophyte nucleomorph genomes sequenced thus far; however, these genomes have a remarkable variation in the number of protein-coding genes (table 1). The nucleomorph genomes of *A. amoebiformis*, *B. natans*, *L. vacuolata*, and *L. oceanica* have 300, 288, 319, and 596 protein-coding genes, respectively (table 1). This variation is mainly caused by duplicated genes; thus, the number of nonredundant protein genes is almost identical among three species except for *L. oceanica* (295, 288, 294, and 338, respectively). Approximately 60% of nucleomorph-encoded proteins are annotated by homology with sequences of other organisms, and 40% are nucleomorph-specific hypothetical proteins, the so-called ORFans, that have no sequence similarity with any genes in databases.

**Fig. 3.—:**
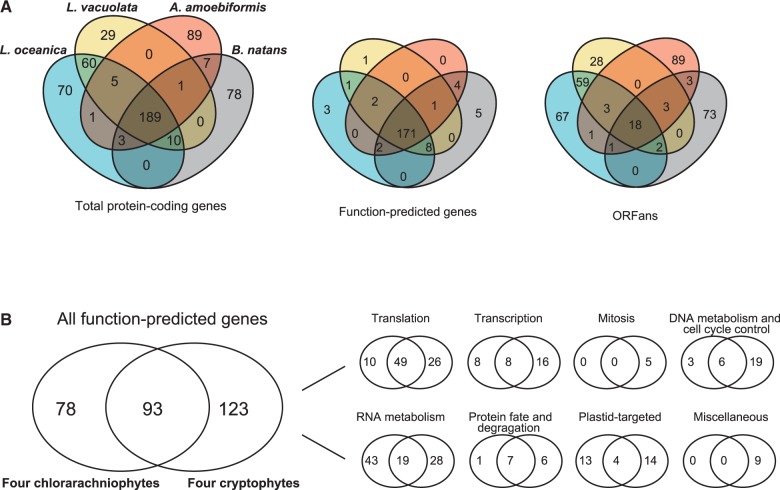
Comparison of gene content among nucleomorph genomes. (*A*) Comparison of gene content among four chlorarachniophyte nucleomorph genomes. Venn diagrams indicate the number of shared and/or unique genes categorized as total protein-coding genes, function-predicted protein genes, and hypothetical protein genes (ORFans). (*B*) Comparison of conserved core genes between four chlorarachniophytes and four cryptophytes. Total 93 function-predicted genes are overlapped among eight nucleomorph genomes of chlorarachniophytes and cryptophytes. Light Venn diagrams show the number of shared and/or unique genes in each functional category.

The nucleomorph genomes of four chlorarachniophytes, *A. amoebiformis*, *B. natans*, *L. vacuolata*, and *L. oceanica,* share 189 protein-coding genes, including 171 function-predicted genes and 18 ORFans (fig. 3*A* and supplementary table S1, Supplementary Material online). Total 198 function-predicted genes have been annotated in chlorarachniophyte nucleomorph genomes, and 86% (171/198) of them are overlapped among four genomes (fig. 3*A*). In cryptophytes, 69% (216/311) function-predicted genes are shared among four nucleomorph genomes (Moore et al. 2012), suggesting that chlorarachniophyte nucleomorph genomes are less diverse than cryptophyte ones in term of gene content. Interestingly, all four chlorarachniophyte nucleomorphs possess the same set of genes encoding 17 plastid-associated proteins. The other annotated genes mainly encode housekeeping proteins for transcription, translation, DNA/RNA metabolism, and protein folding/degradation, and these genes would remain in the nucleomorph genomes for expression of the 17 plastid-associated proteins. When content of nucleomorph conserved core genes in four chlorarachniophytes was compared with those in four cryptophytes, 93 of 171 chlorarachniophyte core genes (54%) were overlapped with the cryptophyte core genes (fig. 3*B*). Shared genes in the both groups were found in multiple categories of eukaryotic housekeeping functions (e.g., translation, transcription, DNA/RNA metabolism, and protein fate and degradation), and half of shared genes (49/93 genes) were categorized as translation (fig. 3*B*). These data suggest that there are similar reductive pressures on nucleomorph-retained genes in both chlorarachniophytes and cryptophytes.

Although annotated genes are mostly conserved among the four nucleomorph genomes of chlorarachniophytes, several genes have been lost independently in each species. For instance, *A. amoebiformis*, *B. natans*, *L. vacuolata*, and *L. oceanica* lacked 18, 7, 14, and 11 annotated genes, respectively. Tanifuji et al. (2014) surveyed recent gene transfers of nucleomorph missing genes from nucleomorph to nuclear genomes by using the nuclear genome and transcriptome data of *B. natans* and *L. oceanica*; however, no evidence of gene transfer was found. We also searched for the lineage-specific nucleomorph missing genes in the nuclear genome of *B. natans* and the transcriptome data of *A. amoebiformis*, but did not detect any of them. The nucleomorph-to-nucleus gene transfer presumably did not occur after the divergence of chlorarachniophyte species. One possible explanation is that the difference in GC content between nucleomorph and nuclear genomes (average 29% and 45%, respectively, in *B. natans*) would be a barrier for the expression of transferred genes and successful gene transfer. Overall, these data suggest that chlorarachniophyte nucleomorph genomes would have almost reached an end point in reductive evolution; however, they maintain some room for further reduction. Although conserved genes among different chlorarachniophyte nucleomorphs have been mostly annotated by homology searches, many hypothetical protein-coding genes (ORFans) are found to be lineage-specific genes. Even when closely related *Lotharella* species are compared, they have 59 lineage-specific ORFans (52.7% and 39.1% of the total ORFans in *L. vacuolata* and *L. oceanica*, respectively) (fig. 2 and supplementary table S1, Supplementary Material online). This suggests that loss and gain of many ORFans occurred independently after the divergence of chlorarachniophyte species. The function of ORFans is unclear, and it is hypothesized that ORFans may have taken over the function of lineage-specific missing genes such as those described above (Tanifuji et al. 2014).

It has been reported that the nucleomorph genome of *B. natans* has two permuted tRNA^Ser^ genes, *trnS* (AGA) and *trnS* (CGA), which have also been found in the nuclear genomes of several green algae, including *Ostreococcus* and *Micromonas* (Maruyama et al. 2010). We found those two permuted tRNA^Ser^ genes in the *A. amoebiformis* nucleomorph genome, but no permuted tRNA was detected in *L. vacuolata* and *L. oceanica*. Thus, the green algal ancestor of chlorarachniophyte plastids is postulated to have permuted tRNA^Ser^ genes; however, *L. vacuolata* and *L. oceanica* would have lost these genes after the divergence of chlorarachniophyte species.

The four nucleomorph genomes of chlorarachniophytes lack 5S rRNA gene, which is common in cryptophyte nucleomorph genomes. It has been known that yeast 5S rRNA recruits two ribosomal proteins, Rpl5 and Rpl11, to form 5S ribonucleoprotein particle, which is incorporated into eukaryotic 60S preribosomes (Staley and Woolford 2009), and the C-terminal basic region of Rpl5 is important in the binding to 5S rRNA (Deshmukh et al. 1995). Although homologous genes for Rpl5 and Rpl11 were found in nucleomorph genomes of all four chlorarachniophytes, but the C-terminal regions of Rpl5 were highly divergent compared with homologs of other organisms. Additionally, several genes for PPC-targeted 60S ribosome components are absent from both nucleomorph and nuclear genomes in the chlorarachniophyte *B. natans*, whereas almost complete set of PPC-targeted ribosome genes have been found in genomes of the cryptophyte *G. theta* (Curtis et al. 2012). Sequence divergence of the key ribosomal protein and partially lacking of 60S ribosome components might be related to the missing 5S rRNA gene in chlorarachniophyte nucleomorph genomes.

### Ultrasmall Introns of Nucleomorph Genes

**Fig. 4.—:**
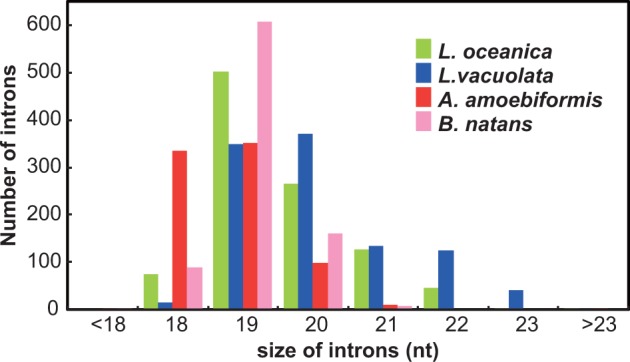
Size distribution of ultrasmall introns in chlorarachniophyte nucleomorph genes. The total number of introns in each size category is indicated by color bars: *B. natans* (pink), *A. amoebiformis* (red), *L. vacuolata* (blue), and *L. oceanica* (green).

The chlorarachniophyte nucleomorph genomes are known to have numerous ultrasmall spliceosomal introns ranging from 18 to 23 nt (865 and 1,021 in *B. natans* and *L. oceanica*, respectively), whereas cryptophyte nucleomorphs have a small number of introns (0–24) (Lane et al. 2007; Moore et al. 2012). In this study, we predicted 793 and 1,028 of ultrasmall introns in *A. amoebiformis* and *L. vacuolata*, respectively (table 1). Most of these introns are 18–23 nt in size and possess a typical spliceosomal boundary (5′-GT and AG-3′), which is similar to that observed in other chlorarachniophytes (fig. 4). A remarkable difference of introns among four chlorarachniophyte nucleomorph genomes is the size distribution (fig. 4). The proportion of 19-nt introns is the highest in *B. natans* (70.3%) and *L. oceanica* (49.3%), whereas 18- and 20-nt introns are abundant in *A. amoebiformis* (42.1%) and *L. vacuolata* (35.8%), respectively. Total intron sizes are 14.9, 16.5, 20.0, and 20.9 kb in *A. amoebiformis*, *B. natans*, *L. oceanica*, and *L. vacuolata*, respectively. A positive correlation between the nucleomorph genome size and the intron length was assumed (Tanifuji et al. 2014), but our data do not support this hypothesis. Although most of the introns are 18–23 nt in size, we found two exceptional introns that were 40 and 41 nt in size in the different positions of *A. amoebiformis* and *L. vacuolata prp43-2* genes, respectively (supplementary fig. S1, Supplementary Material online). These introns could be derived from the fusion of two ultrasmall introns because a relict AG boundary exists at the center of the introns. *Lotharella oceanica prp43-2* also has a 32-nt intron (Tanifuji et al. 2014) at the same position as that of the 40-nt intron in *A. amoebiformis*, which would be the result of size reduction, following the intron fusion.

It has been reported that the positions of ultrasmall introns are mostly conserved among homologous genes of *B. natans*, *L. oceanica*, and *Gymnochlora stellata* (Slamovits and Keeling 2009; Tanifuji et al. 2014). We compared 290 introns within 55 conservative homologous genes among four chlorarachniophyte nucleomorph genomes. The positions of 38.3% introns were identical in the four species, and 86.6% introns were conserved in at least two species (supplementary table S2, Supplementary Material online). Many ultrasmall introns were established in the current style before the divergence of chlorarachniophytes, and lineage-specific intron gain and loss seems infrequent.

In terms of splicing machinery for nucleomorph transcripts, we identified several spliceosomal protein genes and three snRNA genes (*U2*, *U5,* and *U6*) in the *A. amoebiformis* and *L. vacuolata* nucleomorph genomes. The *B. natans* nucleomorph genome encodes *U1*, *U2*, *U5*, and *U6* snRNA, and the *L. oceanica* nucleomorph genome has *U2*, *U5*, and *U6* snRNA genes. Tanifuji et al. (2014) reported that the *L. oceanica* nucleomorph completely lacked all of the snRNAs, but our survey detected three snRNAs. The *U4* snRNA is absent from all nucleomorph genomes, which is consistent with the ultrasmall size of introns, because *U4* snRNA is generally used to bring two remote splice sites together (Staley and Guthrie 1998). *U1* snRNA has a function to identify a 5′ splice site, but three nucleomorph genomes unexpectedly lack this gene. It is likely that we simply could not find several snRNA genes due to the low sequence similarity with canonical snRNAs. However, we could not exclude the possibility that snRNAs are transported from the nucleus to the nucleomorph across multiple plastid envelope membranes.

### Rearrangement of Nucleomorph Genomes

**Fig. 5.—:**
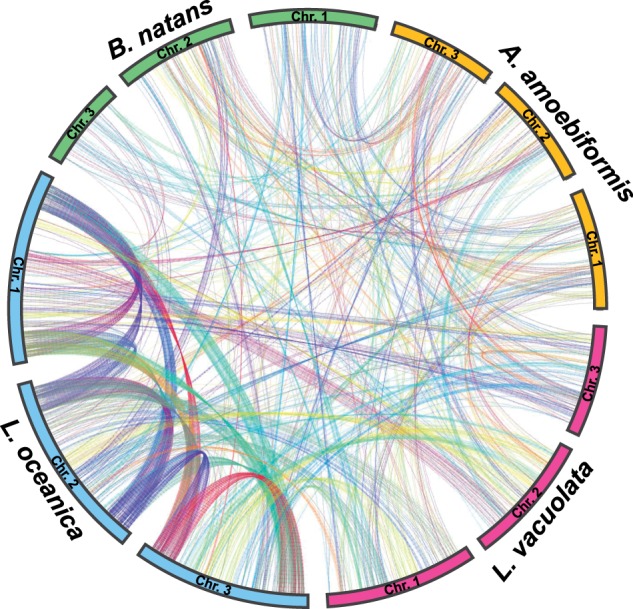
Comparison of homologous gene positions on nucleomorph genomes in four chlorarachniophytes. Colored blocks show three chromosomes in each nucleomorph genome: *B. natans* (green), *A. amoebiformis* (yellow), *L. vacuolata* (red), and *L. oceanica* (blue). The internal lines indicate paired homologous genes. Subtelomeric regions comprised an rDNA operon are excluded from this comparative analysis.

Comparative analyses of nucleomorph genomes have revealed the existence of gene order conservation, so-called synteny, among distantly related species (Moore et al. 2012; Tanifuji et al. 2014). Lane et al. (2007) suggested that nonhomologous recombination events are likely to disrupt coding sequences in extremely reduced and compacted nucleomorph genomes. Therefore, recombination frequency is decreased, resulting in the retention of many syntenic blocks in nucleomorph genomes. In cryptophyte nucleomorphs, the average number of genes within a syntenic block consisting of four or more homologous genes, excluding ORFans, between two of *G. theta*, *H. andersenii*, *Cr. paramecium*, and *Ch. mesostigmatica*, is 6.7–19.4 (Moore et al. 2012). Our comparative analysis of four chlorarachniophyte nucleomorphs indicated that syntenic blocks were composed of 6.2 (*n* = 17), 5.9 (*n* = 13), and 5.8 (*n* = 14) genes between *B. natans* and *A. amoebiformis*, *L. vacuolata*, and *L. oceanica*, 6.5 (*n* = 11) genes between *A. amoebiformis* and *L. vacuolata*, 6.9 (*n* = 11) genes between *A. amoebiformis* and *L. oceanica*, and 11.5 (*n* = 21) genes between *L. vacuolata* and *L oceanica,* on average when the same definition as that used by Moore et al. (2012) was applied (fig. 5 and supplementary fig. S2, Supplementary Material online, and table 3). Nucleomorph genomes appear to be more scrambled in chlorarachniophytes than in cryptophytes. Even when two closely related *Lotharella* species (nucleus- and nucleomorph-encoded small subunit rDNAs are 95% and 99% identical, respectively, between *L. vacuolata* and *L. oceanica*) were compared, approximately 20% of the total genes (61/319 genes) were excluded from syntenic blocks in the *L. vacuolata* nucleomorph genome. Many of the syntenic blocks between *L. vacuolata* and *L. oceanica* are disrupted by duplicated gene regions (fig. 2). These data suggest that genomic rearrangement of chlorarachniophyte nucleomorphs seems to be under progression at the species level, and recombination frequency would be higher in duplicated regions.

**Table 3:** Average Number of Homologous Genes in Syntenic Blocks

	*Bigelowiella natans*	*Amorphochlora amoebiformis*	*Lotharella vacuolata*	*Lotharella oceanica*
*B. natans*	—	6.2 (*n* = 17)	5.9 (*n* = 13)	5.8 (*n* = 14)
*A. amoebiformis*		—	6.5 (*n* = 11)	6.9 (*n* = 11)
*L. vacuolata*			—	11.5 (*n* = 21)
*L. oceanica*				—

**Fig. 6.—:**
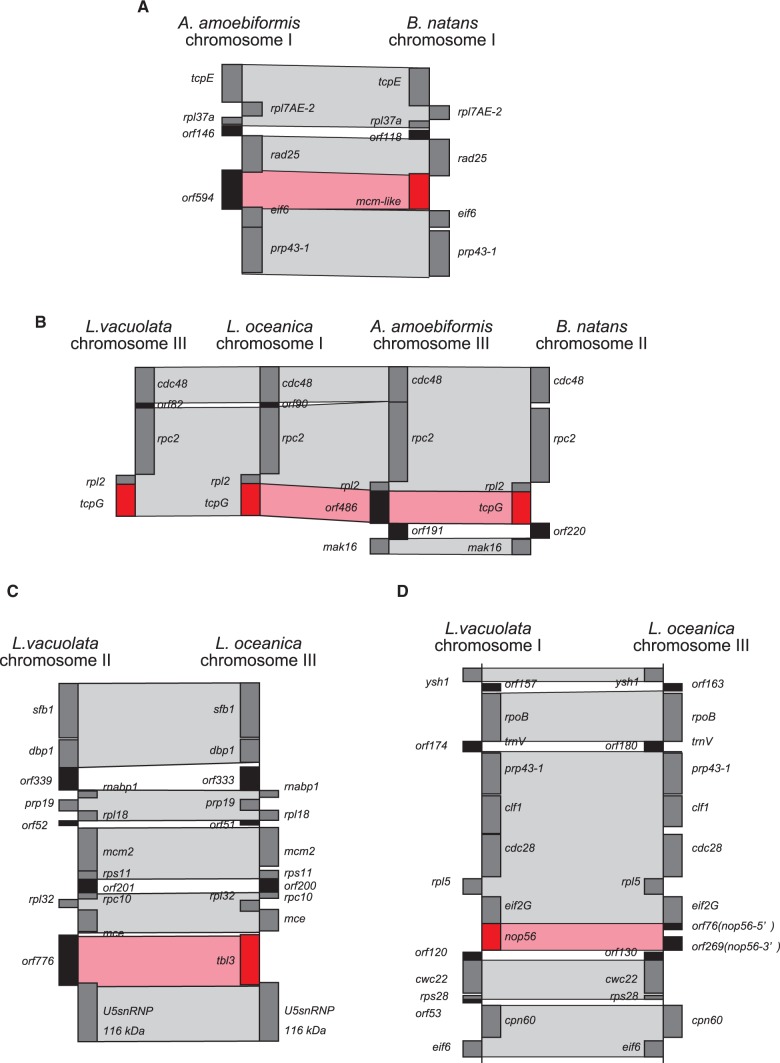
Degraded ORFans in syntenic blocks of chlorarachniophyte nucleomorph genomes. Functions of annotated genes and ORFan genes are shown in gray and black boxes, respectively. Gray highlights indicate syntenic positions between different nucleomorph genomes. Red highlights show the correspondence of syntenic ORFans and functional annotated genes that occupy the same syntenic positions. (*A*) The *mcm-like* gene of *B. natans* corresponds to an ORFan of *A. amoebiformis*. (*B*) *tcpG* genes of three chlorarachniophytes occupy the same syntenic position as that of an ORFan in *A. amoebiformis*. (*C*) The *L. oceanica tbl3* gene corresponds to an ORFan in *L. vacuolata*. (*D*) The *L. vacuolata nop56* gene corresponds to two ORFans in *L. oceanica*.

Nucleomorph syntenic blocks contain several ORFans, the so-called syntenic ORFans, encoding nucleomorph-specific hypothetical proteins. It is difficult to predict the origins and functions of ORFans because of their high sequence diversity. However, syntenic ORFans have the potential for estimating homologous genes through comparison of positions and coding gene sizes among different nucleomorph genomes. In cryptophytes, a portion of ORFans are located at the same syntenic position as functional annotated genes found in the other nucleomorph genomes, suggesting that those ORFans originated by diversification of the annotated genes (Lane et al. 2007; Moore et al. 2012). Our comparative analysis detected several syntenic ORFans in chlorarachniophyte nucleomorph genomes (fig. 6). The 594-amino acid coding ORFan (*orf594*) of *A. amoebiformis* is located between *rad25* and *eif6*, and the *mcm-like* gene composed of 606 amino acids occupies the same syntenic position of *B. natans* (fig. 6*A*). *Amorphochlora amoebiformis* possesses the 486-amino acid ORFan (*orf486*) next to *rpl2*, whereas the other three chlorarachniophytes have *tcpG* genes (480–506 amino acids) at the same position (fig. 6*B*). The *L. vacuolata orf776* between *mce* and *U5 snRNP* (116 kDa) is located in the same syntenic position of *L. oceanica tbl3* (780 amino acids) (fig. 6*C*). These data suggest that nucleomorph-encoded ORFans are generated by sequence divarication of functional annotated protein genes in both chlorarachniophytes and cryptophytes. However, it remains unclear whether ORFans still have the original function of homologous genes or not.

We also found disrupted syntenic ORFans in the *L. oceanica* nucleomorph genome. Two ORFans (*orf76* and *orf269*) of *L. oceanica* occupy the same syntenic position of *L. vacuolata nop56* (414 amino acids) between *eif2G* and *cwc22* (fig. 6*D*). Interestingly, these two ORFans have similarity in the 5′ and 3′ partial coding sequence of *nop56*, and the coding region is divided into two ORFans by a stop codon in the first exon. Similar phenomena have been reported in cryptophyte nucleomorph genomes. For instance, *sf3b3-like* of *Ch. mesostigmatica* corresponds to three syntenic ORFans of *Cr. paramecium*. These data imply another generating system of nucleomorph ORFans by the splitting of gene coding regions. In fact, the average protein size of ORFans (237.2 amino acids) was significantly smaller than that of functional annotated genes (348.6 amino acids, *P* < 0.01, *t*-test) in the chlorarachniophytes.

## Conclusion

Nucleomorph genomes are highly reduced through the achievement of secondary endosymbiosis. In this study, we sequenced two complete nucleomorph genomes of chlorarachniophytes, *A. amoebiformis* and *L. vacuolata*. Our comparative analyses of nucleomorph genomes in four chlorarachniophyte species proposed that most of the functional annotated genes were shared between them, and a small number of core gene losses was observed in each nucleomorph genome individually. This suggests that reductive evolution of the nucleomorph genomes in chlorarachniophytes has mostly reached an endpoint, and that the genome reduction of chlorarachniophyte nucleomorphs has progressed more than that of cryptophytes that are undergoing gene losses associated with core eukaryotic housekeeping functions (Moore et al. 2012). Our data also revealed that size increases of nucleomorph genomes occurred through multiple gene duplications in *Lotharella* species. As found in a previous study, the chlorarachniophyte *Partenskyella glossopodia* is predicted to have an extremely large nucleomorph genome, over 1 Mb in size (Ishida et al. 2011), which is approximately twice as large as the *Lotharella* nucleomorph genomes. To gain further insight into nucleomorph genome expansion, we will sequence the nucleomorph genome of *P. glossopodia* in future studies.

## Supplementary Material

Supplementary tables S1–S4 and figure S1 are available at *Genome Biology and Evolution* online (http://www.gbe.oxfordjournals.org/).

## References

[evv096-B1] Altschul SF 1997 Gapped BLAST and PSI-BLAST: a new generation of protein database search programs. Nucleic Acids Res. 25:3389–3402. 9254694 10.1093/nar/25.17.3389 PMC146917

[evv096-B2] Archibald JM 2007 Nucleomorph genomes: structure, function, origin and evolution. Bioessays 29:392–402. 17373660 10.1002/bies.20551

[evv096-B3] Archibald JM 2009 The puzzle of plastid evolution. Curr Biol. 19:R81–R88. 19174147 10.1016/j.cub.2008.11.067

[evv096-B4] Archibald JM Lane CE 2009 Going, going, not quite gone: nucleomorphs as a case study in nuclear genome reduction. J Hered. 100:582–590. 19617523 10.1093/jhered/esp055

[evv096-B5] Curtis BA 2012 Algal genomes reveal evolutionary mosaicism and the fate of nucleomorphs. Nature 492:59–65. 23201678 10.1038/nature11681

[evv096-B6] del Campo J Not F Forn I Sieracki ME Massana R 2012 Taming the smallest predators of the oceans. ISME J. 7:1–8. 22810060 10.1038/ismej.2012.85 PMC3554395

[evv096-B7] Deshmukh M Stark J Yeh LC Lee JC Woolford JL Jr 1995 Multiple regions of yeast ribosomal protein L1 are important for its interaction with 5 S rRNA and assembly into ribosomes. J Biol Chem. 270:30148–30156. 8530422 10.1074/jbc.270.50.30148

[evv096-B8] Douglas SE 2001 The highly reduced genome of an enslaved algal nucleus. Nature 410:1091–1096. 11323671 10.1038/35074092

[evv096-B9] Douglas SE Murphy C Spencer D Gray M 1991 Cryptomonad algae are evolutionary chimaeras of two phylogenetically distinct unicellular eukaryotes. Nature 350:149–151. 10.1038/350148a0 2005963

[evv096-B10] Douglas SE Penny SL 1999 The plastid genome of the cryptophyte alga, *Guillardia theta*: complete sequence and conserved synteny groups confirm its common ancestry with red algae. J Mol Evol. 48:236–244. 9929392 10.1007/pl00006462

[evv096-B11] Eschbach S Hofmann CJB Maier U-G Sitte P Hansmann P 1991 A eukaryotic genome of 660 kb: electrophoretic karyotype of nucleomorph and cell nucleus of the cryptomonad alga, *Pyrenomonas salina*. Nucleic Acids Res. 19:1779–1781. 2030961 10.1093/nar/19.8.1779 PMC328104

[evv096-B12] Fulnečková J 2012 Dynamic evolution of telomeric sequences in the green algal order Chlamydomonadales. Genome Biol Evol. 4:248–264. 22247428 10.1093/gbe/evs007 PMC3318450

[evv096-B13] Gilson PR 2006 Complete nucleotide sequence of the chlorarachniophyte nucleomorph: Nature’s smallest nucleus. Proc Natl Acad Sci U S A. 103:9566–9571. 16760254 10.1073/pnas.0600707103 PMC1480447

[evv096-B14] Gilson PR McFadden GI 1999 Molecular, morphological and phylogenetic characterization of six chlorarachniophyte strains. Phycol Res. 47:7–19.

[evv096-B15] Gould SB Waller RF McFadden GI 2008 Plastid evolution. Annu Rev Plant Biol. 59:491–517. 18315522 10.1146/annurev.arplant.59.032607.092915

[evv096-B16] Hibberd D Norris R 1984 Cytology and ultrastructure of *Chlorarachnion reptans* (Chlorarachniophyta divisio nova, Chlorarachniophyceae classis nova). J Phycol. 20:310–330.

[evv096-B17] Hirakawa Y Ishida K 2014 Polyploidy of endosymbiotically derived genomes in complex algae. Genome Biol Evol. 6:974–980. 24709562 10.1093/gbe/evu071 PMC4007541

[evv096-B18] Hopkins JF 2012 Proteomics reveals plastid- and periplastid-targeted proteins in the chlorarachniophyte alga *Bigelowiella natans*. Genome Biol Evol. 4:1391–1406. 23221610 10.1093/gbe/evs115 PMC3542566

[evv096-B19] Ishida K 2005 Protein targeting into plastids: a key to understanding the symbiogenetic acquisitions of plastids. J Plant Res. 118:237–245. 16044198 10.1007/s10265-005-0218-2

[evv096-B20] Ishida K Cao Y Hasegawa M Okada N Hara Y 1997 The origin of chlorarachniophyte plastids, as inferred from phylogenetic comparisons of amino acid sequences of EF-Tu. J Mol Evol. 45:682–687. 9419245 10.1007/pl00006272

[evv096-B21] Ishida K Endo H Koike S 2011 *Partenskyella glossopodia* (Chlorarachniophyceae) possesses a nucleomorph genome of approximately 1 Mbp. Phycol Res. 59:120–122

[evv096-B22] Ishida K Green B Cavalier-Smith T 1999 Diversification of a chimaeric algal group, the chlorarachniophytes: phylogeny of nuclear and nucleomorph small-subunit rRNA genes. Mol Biol Evol. 16:321–331.

[evv096-B23] Kasai F Kawachi M Erata M Yumoto K Sato M 2009 NIES-collection list of strains. 8th ed. Jpn J Phycol (Sosui). 57:220.

[evv096-B24] Keeling PJ 2010 The endosymbiotic origin, diversification and fate of plastids. Philos Trans R Soc B Biol Sci. 365:729–748. 10.1098/rstb.2009.0103 PMC2817223 20124341

[evv096-B25] Keeling PJ 2014 The Marine Microbial Eukaryote Transcriptome Sequencing Project (MMETSP): illuminating the functional diversity of eukaryotic life in the oceans through transcriptome sequencing. PLoS Biol. 12:e1001889. 24959919 10.1371/journal.pbio.1001889 PMC4068987

[evv096-B26] Kin T 2007 fRNAdb: a platform for mining/annotating functional RNA candidates from non-coding RNA sequences. Nucleic Acids Res. 35:D145–D148. 17099231 10.1093/nar/gkl837 PMC1669753

[evv096-B27] Lagesen K 2007 RNAmmer: consistent and rapid annotation of ribosomal RNA genes. Nucleic Acids Res. 35:3100–3108. 17452365 10.1093/nar/gkm160 PMC1888812

[evv096-B28] Lane CE Archibald JM 2006 Novel nucleomorph genome architecture in the cryptomonad genus *Hemiselmis*. J Eukaryot Microbiol. 53:515–521. 17123416 10.1111/j.1550-7408.2006.00135.x

[evv096-B29] Lane CE 2007 Nucleomorph genome of *Hemiselmis andersenii* reveals complete intron loss and compaction as a driver of protein structure and function. Proc Natl Acad Sci U S A. 104:19908–19913. 18077423 10.1073/pnas.0707419104 PMC2148396

[evv096-B30] Maruyama S Sugahara J Kanai A Nozaki H 2010 Permuted tRNA genes in the nuclear and nucleomorph genomes of photosynthetic eukaryotes. Mol Biol Evol. 27:1070–1076. 20022888 10.1093/molbev/msp313

[evv096-B31] Moore CE Curtis B Mills T Tanifuji G Archibald JM 2012 Nucleomorph genome sequence of the cryptophyte alga *Chroomonas mesostigmatica* CCMP1168 reveals lineage-specific gene loss and genome complexity. Genome Biol Evol. 4:1162–1175. 23042551 10.1093/gbe/evs090 PMC3514955

[evv096-B32] Moore D Dowhan D Chory J Ribaudo RK 2002 Isolation and purification of large DNA restriction fragments from agarose gels. Curr Protoc Stem Cell Biol. 59:2 6.1–2.6.12. 10.1002/0471142727.mb0206s59 18265307

[evv096-B33] Palmer JD 1997 Organelle genomes: going, going, gone! Science 275:790–791.

[evv096-B34] Phipps KD Donaher NA Lane CE Archibald JM 2008 Nucleomorph karyotype diversity in the freshwater cryptophyte genus *Cryptomonas*. J Phycol. 44:11–14. 10.1111/j.1529-8817.2007.00434.x 27041033

[evv096-B35] Price DC 2012 *Cyanophora paradoxa* genome elucidates origin of photosynthesis in algae and plants. Science 335:843–847. 22344442 10.1126/science.1213561

[evv096-B36] Rensing SA Goddemeier M Hofmann CJ Maier U-G 1994 The presence of a nucleomorph *hsp70* gene is a common feature of Cryptophyta and Chlorarachniophyta. Curr Genet. 26:451–455. 7874738 10.1007/BF00309933

[evv096-B37] Rodríguez-Ezpeleta N 2005 Monophyly of primary photosynthetic eukaryotes: green plants, red algae, and glaucophytes. Curr Biol. 15:1325–1330. 16051178 10.1016/j.cub.2005.06.040

[evv096-B38] Rogers MB Gilson PR Su V McFadden GI Keeling PJ 2007 The complete chloroplast genome of the chlorarachniophyte *Bigelowiella natans*: evidence for independent origins of chlorarachniophyte and euglenid secondary endosymbionts. Mol Biol Evol. 24:54–62. 16990439 10.1093/molbev/msl129

[evv096-B39] Rutherford K 2000 Artemis: sequence visualization and annotation. Bioinformatics 16:944–945. 11120685 10.1093/bioinformatics/16.10.944

[evv096-B40] Schattner P Brooks AN Lowe TM 2005 The tRNAscan-SE, snoscan and snoGPS web servers for the detection of tRNAs and snoRNAs. Nucleic Acids Res. 33:W686–W689. 15980563 10.1093/nar/gki366 PMC1160127

[evv096-B41] Silver TD 2007 Phylogeny and nucleomorph karyotype diversity of chlorarachniophyte algae. J Eukaryot Microbiol. 54:403–410. 17910684 10.1111/j.1550-7408.2007.00279.x

[evv096-B42] Silver TD Moore CE Archibald JM 2010 Nucleomorph ribosomal DNA and telomere dynamics in chlorarachniophyte algae. J Eukaryot Microbiol. 57:453–459. 21040099 10.1111/j.1550-7408.2010.00511.x

[evv096-B43] Slamovits CH Keeling PJ 2009 Evolution of ultrasmall spliceosomal introns in highly reduced nuclear genomes. Mol Biol Evol. 26:1699–1705. 19380463 10.1093/molbev/msp081

[evv096-B44] Soma A 2007 Permuted tRNA genes expressed via a circular RNA intermediate in *Cyanidioschyzon merolae*. Science 318:450–453. 17947580 10.1126/science.1145718

[evv096-B45] Staley JP Guthrie C 1998 Mechanical devices of the spliceosome: motors, clocks, springs, and things. Cell 92:315–326. 9476892 10.1016/s0092-8674(00)80925-3

[evv096-B46] Staley JP Woolford JL 2009 Assembly of ribosomes and spliceosomes: complex ribonucleoprotein machines. Curr Opin Cell Biol. 21:109–118. 19167202 10.1016/j.ceb.2009.01.003 PMC2698946

[evv096-B47] Sugahara J Yachie N Sekine Y Soma A 2006 SPLITS: a new program for predicting split and intron-containing tRNA genes at the genome level. In Silico Biol. 6:411–418. 17274770

[evv096-B48] Tanifuji G 2011 Complete nucleomorph genome sequence of the nonphotosynthetic alga *Cryptomonas paramecium* reveals a core nucleomorph gene set. Genome Biol Evol. 3:44–54. 21147880 10.1093/gbe/evq082 PMC3017389

[evv096-B49] Tanifuji G 2014 Nucleomorph and plastid genome sequences of the chlorarachniophyte *Lotharella oceanica*: convergent reductive evolution and frequent recombination in nucleomorph-bearing algae. BMC Genomics 15:374. 24885563 10.1186/1471-2164-15-374 PMC4035089

[evv096-B50] Tanifuji G Onodera NT Hara Y 2010 Nucleomorph genome diversity and its phylogenetic implications in cryptomonad algae. Phycol Res. 58:230–237.

[evv096-B51] Wang Y 2012 MCScanX: a toolkit for detection and evolutionary analysis of gene synteny and collinearity. Nucleic Acids Res. 40:e49 22217600 10.1093/nar/gkr1293 PMC3326336

